# An Investigation of the Slope Parameters of Reaction Times and P3 Latencies in the Sternberg Memory Scanning Task – A Fixed-Links Model Approach

**DOI:** 10.5334/joc.158

**Published:** 2021-04-29

**Authors:** Henrike M. Jungeblut, Dirk Hagemann, Christoph Löffler, Anna-Lena Schubert

**Affiliations:** 1 Institute of Psychology, Heidelberg University, Heidelberg, Germany

**Keywords:** Intelligence, Sternberg Memory Scanning Task, Reaction Times, P3 Latencies, Fixed-Links Model, Mental Speed

## Abstract

The speed of short-term memory scanning is thought to be captured in the slope of the linear function of mean reaction times (RTs) regressed on set size in the Sternberg memory scanning task (SMST). Individual differences in the slope parameter have been hypothesized to correlate with general intelligence (*g*). However, this correlation can usually not be found. This present study chose a fixed-links model (FLM) approach to re-evaluate the RT slope parameter on a latent level in a sample of 102 participants aged 18 to 61 years who completed the SMST with set sizes 1, 3, and 5. The same was tried for P3 latencies to investigate whether or not both parameters measure the same cognitive processes in the SMST, and to assess the usability of both slopes to predict *g*. For RTs, a linear increase with set size was found. The slope of mean RTs correlated with *g* on a manifest level already. The FLM approach could better reveal this relationship with the correlation between the slope and *g* being substantially higher. For P3 latencies, we found no evidence for a linear increase, but a general increase from the smallest set size to the two larger ones. This indicates that RTs and P3 latencies do not measure the same cognitive processes in the SMST. The FLM proved suitable to investigate the association between the speed of short-term memory scanning and intelligence.

## Introduction

The search for neuro-cognitive processes that are the basis of intelligence and that differ in individuals of higher and lower general intelligence (*g*) is ongoing in the field of differential psychology. The speed of information processing has been proposed as one underlying general feature (Jensen, 2006). In the present study, we focused on the speed of short-term memory scanning as the feature of the memory scanning process that contributes to interindividual differences in intelligence that, among other processes, gives rise to a single *g*-factor. The goal of the following analyses was to answer the question whether reaction times (RTs) and P3 latencies of the event-related potential (ERP) both measure short-term memory scanning by regressing RTs and P3 latencies on the varying set size conditions in the Sternberg memory scanning task (SMST; Sternberg, 1966). The slopes of the resulting linear functions were then used to predict intelligence. This allows to see whether the speed of short-term memory scanning differs between individuals of higher and lower general intelligence. To do this, the fixed-links model (FLM; introduced by Schweizer, 2006) as a special form of structural equation modeling (SEM) was chosen because earlier research has often failed to correlate the slope parameter with intelligence on a manifest level (Jensen, 1987, 1998).

In the SMST, participants see a set of numbers (called memory set or positive set) varying in set size. They are asked to memorize the memory set displayed. After a short amount of time, the memory set disappears and, after another short delay, a probe stimulus is presented on the screen. Participants then must decide as quickly as possible whether the test stimulus belongs to the positive set or not (and thus belongs to the so-called negative set). Many authors have shown that RTs in this task increase linearly with set size (e.g., Chiang & Atkinson, 1976; Ergen et al., 2012; Jensen, 1987; Pelosi et al., 1995; Sternberg, 1969). Sternberg (1969) offered a psychological explanation for both the slope and the intercept of the linear function of RTs regressed on set size. He interpreted the intercept of the linear function as the time needed for all processes that happen regardless of the number of digits in the memory set. These processes include, for example, the encoding of the test stimulus as well as the preparation and execution of the motor response. The slope of the linear function is thought to represent the additional time needed for the specific process that happens for each member of the positive set. More precisely, this specific process includes a comparison between each member of the memory set and the test stimulus, in addition to a switch from one member to the next. Therefore, the number of digits in the positive set determines the number of comparison operations executed. A stepwise linear increase in set size thus leads to a stepwise linear increase in comparison operations that need to be executed. A linear function captures the resulting increase in RTs. The specific process associated with the slope of this linear function is what Sternberg (1969) called serial memory scanning. In other words, the time needed for this specific process reflects the time one needs to withdraw information (the memory set) from short-term memory.

RTs in the SMST have repeatedly been shown to be negatively correlated with intelligence. In other words, more intelligent individuals show shorter RTs in the memory task. A meta-analysis by Sheppard and Vernon (2008) found a mean correlation of *r* = –.25 between intelligence and RTs in the SMST and similar tasks that resemble the SMST. Similarly, McGarry-Roberts et al. (1992) found a correlation of similar size, *r* = –.21, between RTs in the SMST and general intelligence measured with the Wechsler Adult Intelligence Scale-Revised (WAIS-R; Wechsler, 1981). This negative correlation between RTs and intelligence is not exclusive for the SMST. Several reviews and meta-analyses have shown a consistent negative association between RTs in different simple and choice RT tasks and intelligence. Sheppard and Vernon (2008) found a negative relationship of *r* = –.24 across 172 studies between RTs in various RT tasks and intelligence in their review. A meta-analysis investigating the worst performance rule, which describes the phenomenon that an individual’s slowest response in an RT task is more predictive of their intelligence than an average or fast response, found a correlation of *r* = –.28 between mean RTs and cognitive abilities (Schubert, 2019). Likewise, Doebler and Scheffler (2016) found correlations in the Hick task ranging from *r* = –.18 to *r* = .28 in their meta-analysis.

The two general findings that RTs increase in a linear fashion with increasing set size and that shorter RTs are associated with higher general intelligence, led researchers to use the slope parameter of the linear function to predict individual differences in intelligence. A lower slope should therefore be associated with higher general intelligence and vice versa. However, this hypothesized correlation cannot be consistently confirmed in the literature (Jensen, 1987, 1998). Even if a correlation is found, it is usually small and insignificant (e.g., Neubauer et al., 1997). In addition to the small size of this correlation, mean RTs themselves tend to correlate much higher with intelligence than the slope parameter does. Consequently, this led researchers to abolish the idea of using the slope to predict individual difference in intelligence. Jensen (1992) explained that the reason for a poor prediction of *g* using the slope lies in a statistically built-in negative correlation between the slope and the intercept of the linear function. It is this negative correlation that suppresses any correlation of the slope with other constructs like intelligence. The intercept therefore becomes a suppressor variable that impedes an association between the slope and intelligence. This negative correlation seems theoretically paradoxical as it would mean that individuals who are faster at comparing the probe stimulus with each member of the memory set tend to need more time for processes like motor planning and execution. However, the negative association can be explained statistically by the fact that the slope and the intercept share the same measurement errors. Because the shared measurement errors of the slope and the intercept tend to go in opposite directions, a negative correlation results (Jensen, 1998). At the same time, a study by Jensen (1987) reported a much lower reliability of the slope than of the intercept. Carter et al. (1986) explained that a slope obtained by the least squares method can be conceived algebraically as a difference score. Moreover, it is well known that difference scores have a much lower reliability than mean scores (Cohen, 1988). Thus, the low reliability of difference scores directly affects the slope itself. Taken together, these two properties of slope measures (negative correlation of slope and intercept and low reliability of the slope) may account for the typically low associations between the speed of serial memory scanning and intelligence. In order to still use the slope parameter, one has to control for the artificial correlation of intercept and slope and decrease measurement errors (Jensen, 1998).

### Using a Fixed-Links Model Approach to Estimate the Speed of Serial Memory Scanning

Rammsayer et al. (2017) showed that it is possible to tackle both problems (controlling for an artificial correlation between slope and intercept and decreasing measurement errors) using a model with latent variables. In their study, the authors used a FLM approach as first introduced by Schweizer (2006) to investigate the relationship between RTs and *g* in the Hick task. The Hick task (Hick, 1952) is a classical choice RT task in which participants sit in front of an apparatus with up to 8 bulbs lightening up. They must push the button adjacent to the light bulb as quickly as possible. Typically, participants react fastest when there is only one possible light and slowest when all eight response alternatives are possible. The resulting RTs in this task can be described with a linear function of the BITs, the binary logarithm of the number of lights presented. This finding is commonly known as Hick’s Law:

{\rm{RT}}\ =\ a\ +\ b({\rm{lo}}{{\rm{g}}_{\rm{2}}}{\rm{n}}) = a + b({\rm{BITs}}).

The FLM as a special kind of SEM allows the estimation of the variance of a construct on a latent level (in this case the slope of the linear function). Measurement errors can therefore be controlled. Moreover, it allows researchers to decide whether latent variables should stay orthogonal or whether they can correlate with each other. In contrast to conventional SEM, factor loadings in the FLM are not estimated freely but rather fixed with regards to a hypothesized trajectory of the function. Consequently, the FLM can model a latent slope factor that is not correlated with the intercept, and thus, it can implement Jensen’s (1998) demands for future research that uses the slope to predict individual differences in intelligence.

Rammsayer et al. (2017) used variance decomposition to model two latent variables that both represented RT. The first latent variable, denoted the non-experimental latent variable, corresponded to the intercept of the linear model with manifest variables while the other, denoted experimental latent variable, corresponded to the slope. It was assumed that the non-experimental latent variable was not affected by the experimental manipulation and therefore all factor loadings in the FLM were fixed to 1. The experimental latent variable, on the other hand, was thought to be dependent on the experimental manipulation. Therefore, the factor loadings in the FLM were fixed to the expected trajectory of the function. In the case of the Hick task, the authors fixed the factor loadings to 1, 2, and 4, representing the amount of information in the 0, 1, and 2 bit condition of the Hick task. Using this FLM approach, Rammsayer et al. (2017) could show that the experimental latent variable correlated substantially with a correlation of *r* = –.34 with psychometric intelligence measured through three subscales of the Berlin Intelligence Structure test (BIS; Jäger et al., 1997).

The FLM approach has proven to be able to successfully relate the slope parameter (modelled as an experimental latent variable) with intelligence. Therefore, one aim of the present study was to re-evaluate the usability of the slope parameter of the SMST to predict individual differences in intelligence. A FLM approach that accounts for the measurement errors of RTs and controls for an artificial correlation between the intercept and the slope should allow predicting intelligence to an extent that is not possible using a model with manifest variables only. Therefore, we predicted that the correlation between the slope of RTs as a linear function of set size should be more strongly association with intelligence when estimated in the FLM framework than when estimated on the manifest level.

### Assessing the Speed of Neural Information-Processing

Individual differences in the speed of information-processing cannot only be measured as mean RTs, but also as latencies of the event-related potential (ERP). The typical ERP waveform results when a participant performs a task many times while an electroencephalogram (EEG) is recorded. Since the EEG recordings display the brain’s response to an elicited stimulus combined with other activity irrelevant to the task, the EEG is aggregated over many trials to extract only the relevant brain response to the stimulus. The ERP waveform is characterized by several peaks differing in their latency, amplitude, and peak valence. ERP components are usually named after their valence and peak latency or ordinal position, respectively. Here, the term P3 latency refers to the latency of the third positive voltage wave.

As with RTs, there exists a large body of research on the role of ERP latencies in different single and choice RT tasks and their associations with intelligence. In the SMST in particular, P3 latencies seem to be sensitive to the experimental manipulation of the set size as they increase with a larger memory set (e.g., Ergen et al., 2012; Houlihan, 1998; Pelosi et al., 1995; Schubert et al., 2015). This effect seems to be applicable to other RT tasks with increasing difficulty levels across conditions. A study by Euler et al. (2017) revealed an increase in P3 latencies as a function of choice alternatives in the Hick task. Only the increase from the 1 bit (i.e., two choice alternatives) to 2 bit (i.e., four choice alternatives) condition and not the increase from the 0 bit (i.e., single choice) to 1 bit condition reached statistical significance. Furthermore, especially latencies of ERP components associated with stimulus evaluation, memory updating, and response selection that occur later in the stream of information-processing have been shown to be strongly associated with intelligence (Bazana & Stelmack, 2002; Kapanci et al., 2019; McGarry-Roberts et al., 1992; Saville et al., 2015; Schubert et al., 2015; Schubert et al., 2017; Schubert et al., 2018; Schubert et al., 2019; Troche et al., 2015; Troche & Rammsayer, 2009). In particular, some studies have reported negative correlations between intelligence and P3 latencies measured in the SMST (McGarry-Roberts et al., 1992; Schubert et al., 2015; Schubert et al., 2017). Houlihan (1998), however, could not show that P3 latencies in the SMST were correlated with intelligence. The reported correlations ranged from *r* = –.08 to *r* = .11 and none of them reached the level of significance. The inability of this study to find a correlation between intelligence and P3 latencies might be explicable by an attenuation of the relationship because of a range restriction in the intelligence variable (mean IQ = 114, SD = 9.7). Likewise, Euler et al. (2017) did not find an association between P3 latencies and intelligence in the Hick task. Still, the reported correlations were negative throughout all conditions ranging from *r* = –.15 to *r* = –.18, suggesting P3 latencies might be weakly negatively associated with intelligence. Taken together, the findings on P3 latencies are somewhat less consistent that the ones on RTs. Still, it is evident that the general trend (P3 latencies increase with increasing set size and shorter P3 latencies are correlated with higher intelligence) resembles the general findings regarding RTs in the SMST. Therefore, to the degree that P3 latencies can be described as a linear function of set size in a manner similar to RTs, one could assume that P3 latencies also measure serial memory scanning sensu Sternberg (1969). Hence, if the slopes of RTs and ERP latencies in the SMST both reflected the speed of serial memory scanning, they should be positively related.

However, the evidence for converging associations between behavioral and neural measures of processing speed is generally inconsistent. Schubert et al. (2015) reported correlations between RTs and ERP latencies measured in three different experimental tasks including the SMST that ranged from *r* = .46 to *r* = .53, but only considered latencies of the P100, N150, and P200 component in their analyses. Similarly, Euler et al. (2017) reported significant correlations ranging from *r* = .22 to *r* = .44 between decision times in the Hick task (measured as the latency between stimulus presentation and release of a home button) and the latencies of the N200 and P300 component of the ERP. In comparison, McGarry-Roberts et al. (1992) could not find a significant correlation between RTs and P3 latencies in the SMST. Donchin and Coles (1988) pointed out that correlations between RTs and P3 latencies are generally higher if the reaction to the stimulus is correct. However, with small error percentages ranging from 0.10% to 2.78% across the different RT tasks, this should not account for a low correlation between RTs and P3 latencies in McGarry-Roberts et al. (1992). Hence, McGarry-Roberts et al. (1992) concluded that the two measures do probably not reflect the same cognitive process. P3 latencies are interpreted to be independent of all response production processes like motor planning and execution whereas RTs are not (see also McCarthy & Donchin, 1981).

In conclusion, there is inconsistent evidence for a correlation between P3 latencies and RTs. Moreover, this correlation alone cannot prove that both measures reflect the serial memory scanning sensu Sternberg (1969), because intercept measures of both RTs and P3 latencies are affected by a larger number of cognitive processes than just memory scanning. Instead, the trajectories of the linear regressions of RTs and P3 latencies regressed on set size need to be compared since it is the slope in particular that captures the specific process of interest.

We expect the same problems outlined for slope measures of RTs (an artificial correlation between intercept and slope and a low reliability of the slope as summarized by Jensen (1992)) will also apply to P3 latency data. Therefore, a FLM approach should, again, be helpful to assess the true variance of the latent variable that represents the slope, and to fix the correlation of both latent variables representing the P3 latency to zero. If slopes derived from P3 latencies reflect parameters of the same cognitive process as slopes derived from RTs, they should be positively correlated with the speed of serial memory scanning as measured by RTs. Moreover, they should show comparable negative correlations with intelligence, as more intelligent individuals should show a higher speed of serial memory scanning both on a behavioral and on a neural level. In the present study, we therefore use a FLM approach to reanalyze a previously published dataset (Schubert et al., 2017) and to estimate slope measures of serial memory scanning on a behavioral and a neural level to analyze their convergence and their respective associations with intelligence.

## Materials and Methods

### Participants

In the previously published dataset (Schubert et al., 2017), data from *N* = 134 participants was acquired at three different measurement occasions. Here, we only analyzed data from the first two measurement occasions that were separated by approximately four months. Of the *N* = 122 participants who attended both the first and second measurement occasions, a total of *N* = 117 participants (68 females, 49 males) completed both the SMST at the first and the intelligence test at the second measurement occasion. Data of five participants was discarded due to missing intelligence test or P3 latency data. 35 participants were identified as multivariate outliers: Multivariate outliers in the data space of RTs and BIS subscales (*n* = 15) were discarded for all analyses on RTs and general intelligence and multivariate outliers in the data spaces of P3 latencies and BIS subscales (*n* = 30) were discarded for all analyses of P3 latencies and general subscales (see subsection on data analysis for more information on multivariate outlier detection). *N* = 102 participants were included in the final RT dataset (61 females, 41 males). These participants were between 18 and 61 years old (M = 35.82, SD = 13.57). N = 87 participants were included in the final P3 latency dataset (51 females, 36 males). These participants were between the ages of 18 and 59 (M = 35.69, SD = 13.28). The participants were recruited via local newspaper advertisements, announcements on social media platforms, and distribution of flyers in Heidelberg, Germany. Attention was paid to recruit participants with different educational and occupational backgrounds. All participants had normal or corrected to normal vision and no history of mental illness. All participants gave their informed consent form and received €100 and individual feedback about their results as compensation for their participation. The study was conducted in accordance with the Declaration of Helsinki and the protocol was approved by the Ethics Committee of the Faculty of Behavioral and Cultural Studies at Heidelberg University.

### Materials

In the following section, we only report materials relevant to the present study. In the original study, participants completed two additional RT tasks, Raven’s advanced progressive matrices (APM; Raven et al., 1994), and a personality questionnaire. For more information see Schubert et al. (2017) and Kretzschmar et al. (2018).

#### Sternberg Memory Scanning Task

**Figure 1:**
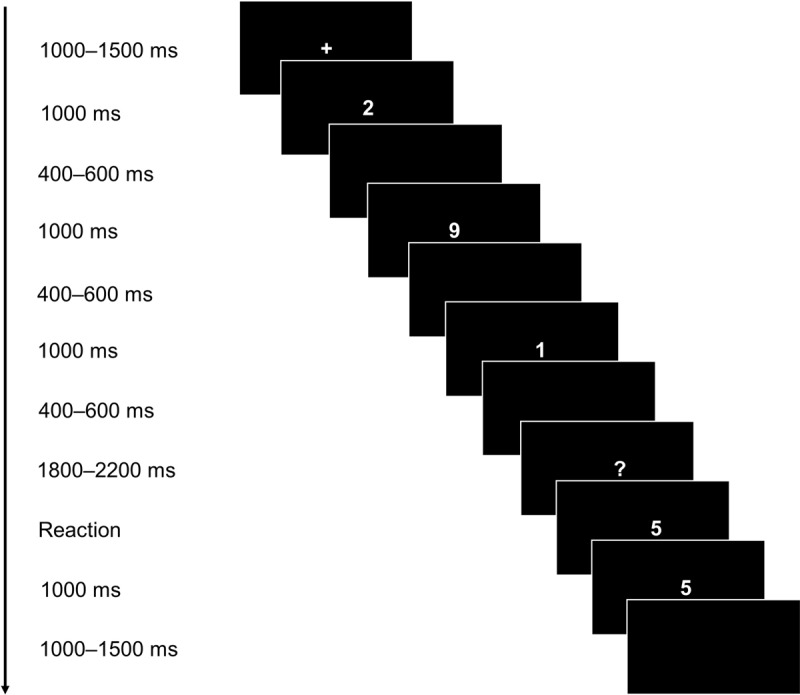
Stimulus Material for the Sternberg Memory Scanning Task. Depicted exemplary for set size 3.

A computer-adapted SMST was used. Participants were shown memory sets consisting of digits between 0 and 9. Three experimental conditions varying in set size were realized (set size 1, 3, and 5). The conditions were presented in a counterbalanced order across participants. A probe stimulus was presented 2000 ms after the memory set. The subjects were instructed to indicate as quickly as possible whether the probe was part of the previously shown positive set or not. Each of the three conditions started with 10 practice trials in which the participants received immediate feedback regarding their performance. The practice trials were followed by 100 test trials without feedback. The stimulus material exemplary for the set size 3 condition is depicted in ***Figure 1***.

Each trial started with the presentation of a white fixation cross in the middle of a black screen. The cross was shown for 1000–1500 ms. The digits of the memory set were presented in a sequential fashion. Each digit was displayed in white on a black screen for 1000 ms with a black screen shown for 400–600 ms between each member of the positive set. Then, after the last digit was presented, a white question mark was shown for 1800–2200 ms. The question mark was followed by the presentation of the probe stimulus. In 50% of all trials, the probe stimulus was part of the memory set. Participants responded pressing one of two keys with their index fingers to indicate whether the probe stimulus was part of the memory set or not. Before the next trial started, the screen remained unchanged for 1000 ms followed by an inter-trial interval of 1000–1500 ms.

#### Berlin Intelligence Structure Test (BIS)

The BIS (Jäger et al., 1997) is based on the hierarchical model of intelligence proposed by Jäger (1982). A single *g* factor stands on the highest hierarchy level. On the second level, Jäger (1982) breaks down general intelligence into the four operation-related components Processing Speed (PS), Processing Capacity (PC), Memory (M), and Creativity (C) and the three content-related levels Figural (F), Numeral (N), and Verbal (V). Each operation-related component is measured on the three content-related levels. In total, the BIS comprises 45 tasks that are all a combination of one operation-related component and one content-related level.

Participants completed the BIS in groups of up to four subjects. Their scores for all seven components were calculated by aggregating the normalized *z*-scores of all tasks belonging to the respective component as suggested by the test manual. Means for all seven components were computed in the dataset of *N* = 117 participants before multivariate outliers were discarded and the dataset split in two. The mean score of the Processing Speed component was M = 97.87 (SD = 7.03), that of the Processing Capacity component was M = 102.75 (SD = 7.49), that of the Memory component was M = 101.73 (SD = 7.95), that of the Creativity component was M = 97.99 (SD = 6.21), that of the Verbal component was M = 102.44 (SD = 6.94), that of the Numerical component was M = 98.24 (SD = 6.60), and that of the Figural component was M = 97.76 (SD = 6.62). These are the mean scores achieved by the participants in the respective component and therefore do not correspond to IQ equivalents. The mean IQ score in our sample was M = 93.53 (SD = 20.30). This is likely an underestimation of participants’ cognitive abilities, as the norm data sample consisted of senior high school students. For our analyses, we subsequently transformed the mean scores of all subscales into *z*-scores. For manifest correlations, *z*-scores from the four subscales PS, PC, M, and C were averaged to form an estimate for *g*. For fixed-links SEMs, we used the four subscales as indicators of a latent general intelligence factor.

### Procedure

Participants completed the SMST among other RT tasks that are not reported here (see Schubert et al., 2017). The BIS and the APM, as well as a personality questionnaire, and questions concerning demographic data were completed at the second measurement occasion. In total, the first measurement occasion took approximately three hours while the second occasion lasted for about three and a half hours. The EEG was recorded during the first measurement occasion while the participants sat in a dimly lit and sound-attenuated cabin and completed the RT tasks.

### EEG Recording

The EEG was recorded with 32 equidistant Ag–AgCl electrodes. The aFz electrode was used as the ground electrode. Electrodes were initially referenced to Cz and offline rereferenced to an average reference. To correct for ocular artefacts, the electrooculogram was recorded bipolarly with two electrodes positioned above and below the left eye and two electrodes positioned at the outer canthi of the eyes. All electrode impedances were kept below 5 kΩ. The EEG signal was recorded continuously with a sampling rate of 1000 Hz (band-pass 0.1–100 Hz), and filtered offline with a low-pass filter of 16 Hz.

### Data Analysis

#### Reaction Time Data

All RTs shorter than 100 ms or longer than 3000 ms were discarded as outliers. Moreover, only RTs of trials in which participants answered correctly were used for further analyses. Logarithmized RTs exceeding ± 3 SDs of the intraindividual mean of each set size condition were also discarded. In total, less than one percent of all RTs were removed. We computed the average across all set size conditions for each participant, which we denoted as mean RT.

#### Electrophysiological Data

We preprocessed the EEG data with the open-source toolbox EEGLAB (Delorme & Makeig, 2004) in MATLAB (The MathWorks Inc., Natick, Massachusetts). ERPs were time-locked to the probe stimulus. The data were filtered with a band-pass filter of 0.1–16 Hz. Epochs had a duration of 1200 ms which included a baseline with a duration of 200 ms before stimulus onset. Bad channels were identified and subsequently removed based on probability and kurtosis of the channel data. Artifact-containing segments were automatically detected and rejected with 1000 μV as the threshold for detecting large fluctuations, 5 SDs as the probability threshold for the detection of improbable data, and 5% as the maximum number of segments to be rejected per iteration. We then conducted independent-component analyses (ICA) of the data down-sampled to 200 Hz and filtered with a high-pass filter of 1 Hz to identify and remove artifacts and generic discontinuities with the ICLabel algorithm (Pion-Tonachini et al., 2019). Subsequently, we repeated the automatic detection and rejection of artifact-containing segments using the same specifications as before and interpolated any previously removed channels. On average, <1% of segments were discarded as outliers or incorrect responses and 12.87% of segments were discarded because they still contained artifacts after IC-based correction. The P3 50% fractional area latency (Luck, 2005) was measured at the parietal electrode over the midline separately for each of the three experimental conditions.

#### Statistical Analysis

After univariate outlier detection a multivariate outlier detection was performed prior to all further analyses. The Mahalanobis distance was calculated for each participant in the data space of RTs, P3 latencies, and BIS subscales resulting in 12 multivariate outliers in the data space of RTs, 27 multivariate outliers in the data space of P3 latencies, and 4 multivariate outliers in the data space of the BIS subscales. For all analyses on RTs, we discarded all multivariate outliers in the dataspaces RTs and BIS subscales (*n* = 15, one participant was identified as outlier in both dataspaces) resulting in a dataset consisting of *N* = 102 participants. Similarly, we discarded all multivariate outliers in the dataspace of P3 latencies and BIS subscales (*n* = 30) for all further analyses on P3 latencies. This resulted in a dataset consisting of *N* = 87 participants.

In a next step, the Shapiro-Wilk and Mardia tests were used to assess the univariate normality in all manifest variables. The R package MVN (Korkmaz et al., 2019) was used for calculating the Mahalanobis distance and to test for deviations from uni- and multivariate normality. The Shapiro-Wilk and Mardia tests showed that the BIS subscales did not deviate significantly from normal distribution. However, RTs and P3 latencies in all set size conditions did (RTs: *p* = .006 for set size 1, *p* < .001 for set size 3, *p* = .033 for set size 5; P3 latencies: *p* < .001 for set size 1, 3, and 5). Nevertheless, deviations from normal distribution of RTs and P3 latencies in all set sizes were regarded as unproblematic because they stayed below threshold values of skewness = 2 and kurtosis = 7, respectively (West et al., 1995).

We used two-factorial repeated measures ANOVAs with the within-subjects factor set size (set size 1 vs. set size 3 vs. set size 5) and the between-subjects factors intelligence (low intelligence vs. high intelligence) to investigate the effects on RTs and P3 latencies, respectively. We used a median split to subdivide the sample into a high intelligence and a low intelligence group. The Greenhouse-Geisser sphericity correction was applied by default. To further investigate the effect of set size on RTs and P3 latencies, we performed post hoc multiple comparisons. We applied the Bonferroni correction to counteract the multiple comparison problem by multiplying *p*-values of post hoc tests by the number of comparisons. To quantify the associations between mean RTs and mean P3 latencies with the averaged BIS subscales, we computed Pearson correlation coefficients. We also regressed RTs on set size for each participant to calculate individual slopes and estimate their correlation with individual differences in *g*. To formally test if RTs and P3 latencies followed the same trajectory, we conducted an additional two-factorial ANOVA with the within-subjects factors set size (set size 1 vs. set size 3 vs. set size 5) and the within-subjects factor measure (RT vs. P3 latency). To see whether age had an effect on our results, we temporarily included age as a second between-subject factor by splitting the group into a high age and a low age group by median split. The ANOVAs were conducted using the statistics software JASP (JASP Team, 2019).

Following the analyses on a manifest level, we used a FLM approach to tackle the problem of low reliability of the slope parameter and to control for the artificial correlation between the intercept and the slope of the linear function. Increasing the reliability of the slope parameter should, in theory, be possible because modeling with latent variables can attain an error-free estimate of the variable of interest (in this case the slope parameter). It should be noted that the FLM was also used to confirm a linear increase of RTs and P3 latencies with increasing set size, as a good model fit for a model that implements the foreseen linear trajectory should be evidence for its linearity.

**Figure 2:**
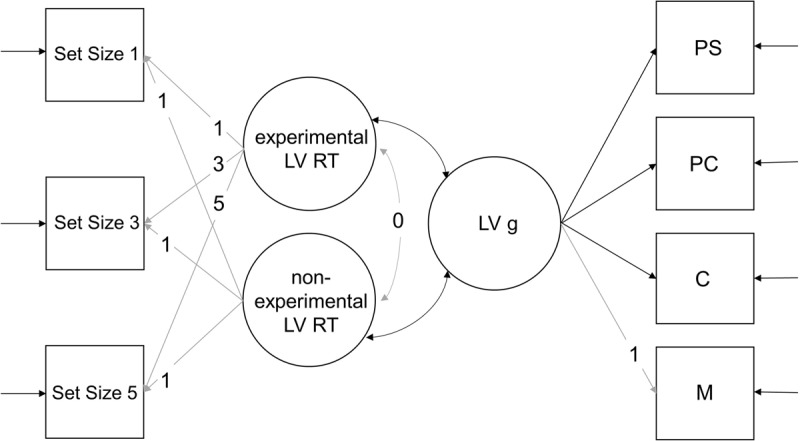
FLM exemplary for RTs. With an experimental latent variable (LV) RT, a non-experimental LV RT, and a LV g. Factor loading of the experimental LV RT were fixed to 1, 3, and 5. Factor loadings of the non-experimental LV RT were fixed to 1. Factor loadings of the LV *g* were estimated freely. The correlation between the experimental and non-experimental LV RT was set to zero, while they were both allowed to correlate with *g* freely. All fixed links are grayed out.

We used the R package lavaan (Rosseel, 2012) to specify structural equation models with the maximum likelihood estimator. We specified a separate model for RTs and P3 latencies, respectively. The full model (exemplary for RTs) is depicted in ***Figure 2***. In both models, RTs and P3 latencies were decomposed into two latent variables identified by three set size specific manifest variables (mean RTs or P3 latencies). The first, denoted non-experimental latent variable, was thought to represent the intercept of the model with manifest variables. We assumed that the non-experimental latent variable was not sensitive to the experimental manipulation of the set sizes and fixed all factor loadings to 1. The second latent variable, denoted experimental latent variable, was thought to represent the slope of the model with manifest variables. The slope, as opposed to the intercept, is thought to be sensitive to the experimental manipulation. Factor loadings were therefore fixed to 1, 3, and 5 to reflect the linear increase of RTs or P3 latencies with increasing set size. Covariances between the two latent variables were fixed to zero to prevent a negative correlation.

In a next step, a latent *g* factor was added to the FLM. This latent variable was measured through the *z*-standardized scores in the four operation-related components PS, PC, M, and C of the BIS. Correlations between the experimental latent variable in the RT or P3 latency model and the latent variable *g* were expected to be bigger than in the model with manifest variables only. As for the manifest model, we wanted to investigate the effects on age on our results. To do so, we temporarily included a regression of the latent variables on age.

Goodness-of-fit was evaluated based on the comparative fit index (*CFI*; Bentler, 1990) and the standardized root mean square residual (*SRMR*; Bentler, 1995). In general, *CFI* values ≥ .90 and *SRMR* values ≤ .10, were considered to indicate an acceptable model fit to the data, while *CFI* values ≥ .95 and *SRMR* values ≤ .05 were considered to indicate a good model fit as suggested by Bentler and Hu (1999). We also conducted the χ^2^-square goodness-of-fit test, however, due to several shortcomings of this test (see Schermelleh-Engel et al., 2003), we only reported the values and did not use it as a formal test statistic. Whenever we tested two competing models against each other, we decided for a model based on Akaike’s information criterion (AIC; Akaike (1973)). A difference Δ between two AIC values <10 indicates that the two models do not substantially differed from each other (Burnham & Anderson, 2002).

For both manifest and latent modeling, a significance level of α = .05 was adopted.

## Results

### Reaction Times and general Intelligence

**Figure 3:**
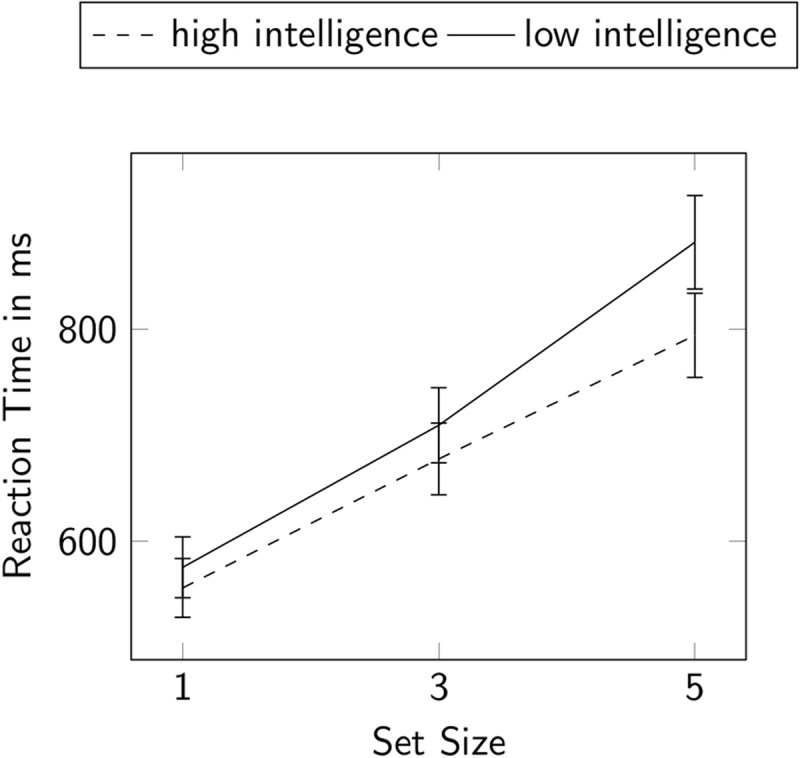
Repeated measures ANOVA for RTs. Participants were split into two groups by median split of general intelligence. Error bars display the 95% confidence interval.

The two-way repeated measures ANOVA (see ***Figure 3***) revealed a significant within-subjects main effect of set size on RTs, *F*(2, 200) = 369.61, *p* < .001, ω^2^ = 0.45, ɛ = 0.74. Post-hoc comparisons showed a significant increase of RTs for each stepwise increase in set size; *t*(101) = –12.75, *p_bonf_* < .001 for the increase from set size 1 to set size 3, and *t*(101) = 14,42, *p_bonf_* < .001 for the increase from set size 3 to set size 5. The ANOVA also yielded a significant main effect of intelligence on RTs, *F*(1, 100) = 4.74, *p* = .032, ω^2^ = .02. The Pearson correlation coefficient yielded a significant negative association between mean RTs and the averaged BIS scales PS, PC, M, and C showing that more intelligent individuals exhibited faster RTs, *r* = –.31, *p* < .001. Furthermore, the ANOVA displayed a significant interaction between set size and intelligence on RTs, *F*(1, 2) = 6.62, *p* = .005, ω^2^ = .01.

The slope of the linear regression for each participant was significantly and negatively correlated with the BIS scores, *r* = –.33, *p* < .001, indicating that more intelligent individuals exhibited a flatter slope. We observed a statistically built-in negative correlation of *r* = –.60, *p* < .001 between the slope and the intercept of the linear function. All intercorrelations between the slope, intercept, the averaged BIS-scores, and mean RTs are summarized in ***Table 1***. These findings show that RTs increased with increasing set size, that RTs were negatively correlated with the BIS-scales, and that RTs of less intelligent individuals displayed a steeper trajectory with increasing set size than RTs of more intelligent ones.

**Table 1:** The slope parameter on a manifest level and its correlation with the intercept, averaged BIS-scores, and mean RT.


	SLOPE	INTERCEPT	AVERAGED BIS-SCORES	MEAN RT

Slope	1 (–)	–	–	–

Intercept	–.60*** (<.001)	1 (–)	–	–

Averaged BIS-Scores	–.33*** (<.001)	0.07 (.462)	1 (–)	–

Mean RT	.57*** (<.001)	.32** (.001)	–31** (.001)	1 (–)


*Note*: * *p* < .05, ** *p* < .01, *** *p* < .001, *p*-values are displayed in parentheses.

To exclude the possibility that participants’ age affected the result that the RT trajectories of more and less intelligent individuals differed, we split our participants in a high and low age group by median split and temporarily included this as a second between-subjects factor in our model. Age did not influence this interaction effect, *F*(2, 200) = 0.73, *p* = .441, ω^2^ = 0.00. Moreover, the main effect of set size on RTs was still significant, *F*(2, 200) = 378.52, *p* < .001, ω^2^ = 0.46.

In a next step, we used a FLM approach to further validate the linear increase of RTs with increasing set size and to investigate whether fixed-links modeling increased the association between the slope and intelligence. As described earlier, RT was decomposed into two latent variables, one experimental latent variable representing the slope and one nonexperimental latent variable representing the intercept. The model provided an acceptable fit to the data, χ^2^(1) = 2.27, *p* = .132, *CFI* = .99, *SRMR* = .06. The experimental latent variable *RT* displayed a significant variance indicating that RTs increased in a linear fashion with increasing set size. Next, a latent variable *g* was added to the model. Factor loadings of the latent general intelligence factor were estimated freely and the experimental and non-experimental latent variables were allowed to covary freely with *g*. The model provided an acceptable fit to the data, χ^2^(17) = 20.73, *p* = .239, *CFI* = .99, *SRMR* = .06. We observed a negative correlation between the latent slope factor and the latent *g* factor, *r* = .39, *p* = .006. There was not a significant correlation between the latent intercept and intelligence, *r* = .13, *p* = 274.

Again, we wanted to exclude the possibility that age contributed to the negative association between the latent slope factor and the latent *g* factor. We temporarily included a regression of the latent variables in our models on age. This model provided an acceptable fit to the data, χ^2^(22) = 31.19, *p* = .092, *CFI* = .98, *SRMR* = .06. We observed a significant variance of the experimental latent variable RT, *p* < .001, indicating that this variable varied across subjects even after controlling for age. We also still observed a significant, albeit slightly smaller association, between the latent slope and intelligence, *r* = –.33, *p* = .023 but not between the intercept and intelligence, *r* = –.11, *p* = .354.

As predicted, the FLM was able to better reveal the association between the slope and intelligence since the correlation on a latent level (*r* = –.39) was substantially higher than the previously reported partial correlation (*r* = –.33) on a manifest level. Our results suggest that participants’ intelligence was more strongly related to their speed of memory scanning when estimated with the FLM approach than when estimated with the regression slope.

### P3 Latencies and general Intelligence

**Figure 4:**
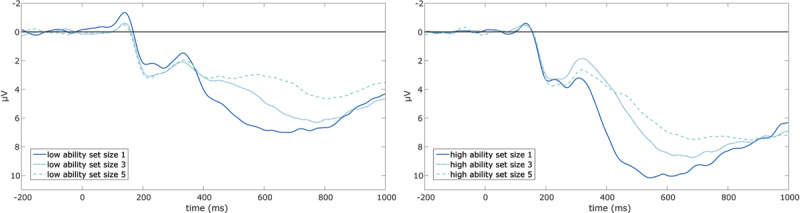
Grand averages of P3 latencies. As measured at the parietal electrode over midline, separately for the low intelligence and the high intelligence group.

**Figure 5:**
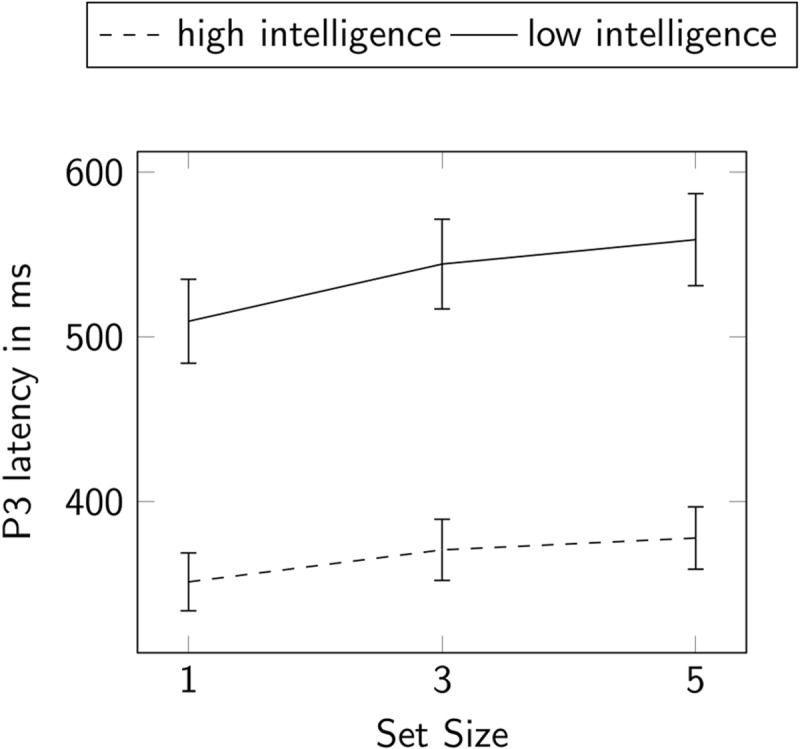
Repeated Measures ANOVA for P3 Latencies. Participants were split into two groups by median split of general intelligence. Error bars display a 95% confidence interval.

Grand-average waveforms of the P3 latencies are presented in ***Figure 4*** separately for more and less intelligent individuals and for each set size condition. On a descriptive level, the grand averages suggested the presence of a main effect of set size on P3 latencies. This was confirmed by the two-way repeated measures ANOVA (see ***Figure 5***) that revealed a significant within-subjects main effect of set size on P3 latencies, *F*(2, 168) = 22.60, *p* < .001, ω^2^ = .01, ɛ = 0.93. Only the increase from set size 1 to 3, *t*(101) = –5.15, *p_bonf_* < .001, and the increase from set size 1 to 5, *t*(101) = –6.32, *p_bonf_* < .001, reached statistical significance while the increase from set size 3 to 5 did not, *t*(101) = –1.18, *p_bonf_* = .725. Still, the results of the ANOVA were not in favor of the hypothesis that P3 latencies increase linearly with set size.

To formally test if RTs and P3 latencies followed the same trajectory, we conducted an additional two-factorial ANOVA with the within-subjects factors set size (set size 1 vs. set size 3 vs. set size 5) and the within-subjects factor measure (RT vs. P3 latency). This revealed a significant interaction effect of the two factors set size and measure, *F*(2, 170) = 126.01, *p* < .001, ω^2^ *= 0.14*. This indicates that P3 latencies follow a different trajectory across set sizes than RTs, suggesting that the two measures do not reflect the same cognitive process. Additionally, there was no significant correlation between mean RTs and P3 latencies which further indicates that RTs and P3 latencies cannot be readily mapped onto each other, *r* = .03, *p* = .795.

Still, a significant between-subjects main effect of intelligence on P3 latencies was found, indicating that more intelligent participants exhibited faster P3 latencies, *F*(1, 84) = 24.15, *p* < .001, ω^2^ = .12. In line with this main effect, the Pearson correlation coefficient unveiled a negative association between mean P3 latencies across all set size conditions and the aggregated BIS scales, *r* = –.37, *p* < .001. Individuals with higher intelligence also showed a shallower increase in P3 latencies with higher set sizes than less intelligent ones, *F*(1, 2) = 3.38, *p* = .040, ω^2^ < .01. Because there was no evidence for a linear increase of P3 latencies with set size, we did not calculate individual slope values to estimate the correlation between the linear increase in P3 latencies across conditions and intelligence.

Like for RTs, we again excluded the possibility that age accounted for our results by temporarily including age as a between-subjects factor in our ANOVA. Age did not have a significant impact on the interaction effect that showed that the trajectories of P3 latencies differed between individuals of higher and lower intelligence, *F*(2, 164) = 2.28, *p* = .106, ω^2^ = 0.00. Moreover, the main effect of set size on RTs was still significant, *F*(2, 164) = 21.25, *p* < .001, ω^2^ = .01.

In a next step, we used the FLM to determine whether a model with factor loadings of the experimental variable fixed to 1, 3, and 5, or alternatively, a model with factor loadings fixed to 1, 3, and 3 that matches the results of the ANOVA best describes our data. We modelled two latent variables for P3 latency: the first denoted non-experimental latent variable P3 representing the intercept, and the second denoted experimental latent variable P3 representing the slope. The first model with factor loading 1, 3, and 5 provided a good fit to the data, χ^2^(1) = 3.88, *p* = .049, *CFI* = .99, *SRMR* = .03, *AIC* = –567.97. Likewise, the model with factor loadings fixed to 1, 3, and 3 provided a good fit to the data, χ^2^(1) = 0.47, *p* = .492, *CFI* = 1, *SRMR* = .01, *AIC* = –571.38. With an AIC difference of Δ = 3.41, we regarded the two models as equally fitting. However, because the ANOVA showed that P3 latencies increased from set size 1 to 3 but not from set size 3 to 5, we decided that the second model with factor loadings of the experimental latent variable fixed to 1, 3, and 3 best represented our data. Because one residual variance parameter of this model was estimated to be negative, we subsequently fixed it to zero. This worsened the model fit only slightly, χ^2^(2) = 6.88, *p* = .032, *CFI* = 99, *SRMR* = .13, *AIC* = –566.97.

Next, we added a latent variable *g* to the FLM. Again, the model provided an acceptable fit to the data, χ^2^(18) = 20.83, *p* = .288, *CFI* = 99, *SRMR* = .09. However, we could not observe a significant correlation between the experimental latent variable P3 and intelligence. Instead, the model yielded a significant relationship between the latent intercept and the latent intelligence variable, *r* = –.32, *p* = .009.

Again, this finding was not any different when controlled for age. We temporarily included a regression of the latent variables on age and observed an acceptable model fit χ^2^(23) = 21.14, *p* = .250, *CFI* = 99, *SRMR* = .08. Similar to the model without age correction, we observed a negative association between the latent intercept and intelligence, *r* = –.42, *p* = .001, but not between the latent slope and intelligence, *p* = .270.

Intelligence was associated with shorter P3 latencies across all set sizes. However, our data could not support the hypothesis that P3 latencies measure the same cognitive processes as RTs. Instead, a model with factor loadings fixed to 1, 3, and 3 best represented our data. The main effect intelligence found in the repeated measures ANOVA was further confirmed by the negative relationship between the latent intercept and the latent variable *g*.

## Discussion

The aim of the present study was to investigate the relationship between the two measures RT and P3 latency in the SMST and to see whether they both measure serial memory scanning sensu Sternberg (1969). Furthermore, the capability of the slope parameter to predict intelligence was re-evaluated using a manifest and a latent variable approach.

### Serial Memory Scanning and Intelligence: Reaction Times

Concerning RTs, the repeated measures ANOVA as well as the FLM supported the hypothesis that RTs increase linearly with set size. This is in line with previous research on the SMST (e.g. Chiang & Atkinson, 1976; Ergen et al., 2012; McGarry-Roberts et al., 1992; Pelosi et al., 1995; Sternberg, 1969). The results agree with Sternberg’s (1969) original interpretation of a serial memory scanning where every member of the memory set has to be compared to the probe stimulus in a single comparison operation, which in turn leads to a linear increase of RTs with every stepwise increase in set size.

Moreover, the present data could show that RTs in the SMST are negatively correlated with the BIS-scores. Former research on the relationship between RTs and intelligence in different RT paradigms (and specifically in the SMST) very much aligns with the present findings (e.g. Doebler & Scheffler, 2016; McGarry-Roberts et al., 1992; Neubauer et al., 1997; Rammsayer et al., 2017; Schubert, 2019; Sheppard & Vernon, 2008).

A negative correlation alone does not tell us a lot about which cognitive process parameters differ between more and less intelligent individuals. By describing RTs as a linear function of set size and thus, decomposing RTs into an intercept (processes like planning and execution of motor response) and a slope (the specific process of comparing every member of the positive set to the probe stimulus), it is possible to only relate the slope parameter to intelligence. In the scope of Sternberg’s (1969) serial memory scanning theory, a negative correlation between the slope and intelligence would indicate that more intelligent individuals profit from faster comparison operations and a faster scanning of the short-term memory, respectively. However, in the literature, the presumed relationship between the slope and intelligence has been difficult to confirm (Jensen, 1987, 1998). Nonetheless, we observed a significant correlation medium size, *r* = –.33, *p* < .001, in the present study. This finding was surprising because we expected the correlation to be rather low or even insignificant. Only a FLM approach was thought to be able to circumvent the problem of low reliability and artificial high correlation between intercept and slope (see Jensen, 1998). Therefore, it needs to be asked why we could find a significant negative correlation of medium size while earlier research often failed to do so. For example, Jensen (1987) found a correlation between the slope of RTs regressed on set size in the SMST and intelligence of *r* = –.06. The same study also investigated the slope of the Hick function and reported a positive correlation of *r* = .19. A possible explanation for these counterintuitive and contradicting results might be a restriction of range of the intelligence variable that was measured with the APM. Jensen (1987) did not report the distribution of the APM scores. However, the sample (*N* = 48) consisted solely of university undergraduates, and thus, it is possible that individuals scoring low on the APM were underrepresented in the sample. A restriction of range in the general intelligence variable would inevitably lead to an attenuated correlation between intelligence and slope. In contrast to the findings of Jensen (1987), Neubauer et al. (1997) did find a negative correlation of *r* = –.17 between the slope of the linear regression and intelligence. Additionally, the sample included participants from different educational and occupational backgrounds. As indicated by the reported mean test scores (M = 23.72, SD = 4.68), it is reasonable to presume that there was no significant range restriction in the intelligence variable in Neubauer et al. (1997). Still, the correlation of *r* = .17 is smaller than the present finding of *r* = –.33. Possibly, different outlier detection procedures that were applied might account for the discrepancy between the correlations. Neubauer et al. (1997) applied a stricter detection criterium with all RTs > 1500 ms discarded that might have led to a restriction of the RT range and consequently to an attenuated variance of the slope. Moreover, participants completed only a fifth (20 per condition) of the number of trials that participants completed in the present study (100 per condition), which must have affected the reliability of the resulting mean RT measures.

Furthermore, there was support for the hypothesis that a FLM approach is indeed able to better reveal the association between the slope and intelligence. The larger correlation between the latent variable RT and intelligence in comparison to the correlation between the manifest slope and intelligence shows that the FLM is a suitable model for investigating the effects of specific processes like serial memory scanning on intelligence. The FLM has also proven to be a suitable model to investigate specific processes in the Hick task as demonstrated by Schweizer (2006) and Rammsayer et al. (2017).

### Serial Memory Scanning and Intelligence: P3 Latencies

As opposed to RTs, it was not possible to find support for the underlying assumption that the P3 latency, interpreted as stimulus evaluation time, reflects serial memory scanning in the sense of Sternberg (1969). P3 latencies increased from set size 1 to set size 3 but then remained stable as shown in the post hoc tests of the repeated measures ANOVA as well as in the FLM with factor loadings fixed to 1, 3, and 3. The inability to demonstrate a linear increase of P3 latencies with set size is inconsistent with Donchin and Coles’s (1988) context updating theory stating that P3 latencies reflect the stimulus evaluation time. On the basis of this theory, it was expected that an increased memory load in larger set sizes would lead to a stepwise increase of the time needed to evaluate the probe stimulus and to decide whether or not it was part of the memory set. Instead, our results are consistent with the idea that P3 latencies reflect the speed of information-transmission from frontal attentional and working memory processes to temporal-parietal processes of memory storage (Polich, 2007; Schubert & Frischkorn, 2020). When participants are only holding a single item in short-term memory, they may not need to recruit these temporal-parietal processes of memory storage. However, as soon as they need to simultaneously hold and search a set of several memory items, they may need to recruit and rely on memory storage processes to update the items held in their focus of attention. It would be interesting to explore this idea using measures of functional connectivity and source analyses, which should reveal an accompanying step-wise increase in connectivity between frontal and temporal-parietal brain regions from set size 1 to larger set sizes. Alternatively, our findings could also be accounted for by theories of stimulus-response reactivation, which propose that the P3 is modulated by stimulus-response bindings (Verleger et al., 2014; Verleger et al., 2017). When comparing the probe stimulus to only a single item held in short-term memory, response preparation may commence as soon as the probe stimulus has been compared to the item held in memory. When comparing the probe stimulus serially to a number of items held in short-term memory, however, already formed stimulus-response bindings may need to be overridden once participants make their decision after reviewing all items held in memory, resulting in longer P3 latencies.

Our results also partially contradict previous studies that found an increase in P3 latencies with set size (e.g. Ergen et al., 2012; Houlihan, 1998; Pelosi et al., 1995; Schubert et al., 2015). Similarly to our findings, however, Pelosi et al. (1995) could only confirm a significant increase of P3 latencies from set size 1 to set size 3. Still, Ergen et al. (2012), Houlihan (1998), and Schubert et al. (2015) reported an overall increase of P3 latencies with set size. Ergen et al. (2012), however, only realized two set size conditions: set size 3 and set size 5. As a consequence, no conclusions concerning the trajectory of P3 latencies in smaller set sizes can be drawn.

The ambiguity between the findings of the aforementioned studies should not be due to the throughout small sample sizes used. A power analysis estimated that the sample size must be at just *N* = 22 to discover a small effect of *f* = .10 in a repeated measures ANOVA given α = .05, 1 – β = .80, and an intercorrelation among the repeated measures of r̅ = .94 (Erdfelder et al., 1996). This indicates that our sample was not underpowered.

Instead, the ambiguity might be explained through the effects of normal ageing on EEG and ERP components as highlighted by Polich (1997). In particular, the P3 component of the ERP was shown to be sensitive to increasing age which is reflected in smaller P3 amplitudes and longer P3 latencies (Polich, 1997). Most of the studies that found an overall increase of P3 latencies acquired data from participants that were considerably younger than in the present study (M = 35.9, SD = 13.5). For example, Ergen et al. (2012) recruited participants with a mean age of 26 years (SD = 5.1). Similarly, the mean age in Houlihan (1998) was 22.7 years (aged 18 to 37 years). This suggests that earlier research may have found an overall increase in P3 latencies because the mean age in the samples was low.

Taken together, our results indicate that neural processing as measured by P3 latencies may reflect a different cognitive process than serial memory scanning, as we found no evidence that P3 latencies increased linearly with set size. The conclusion that behavioral and electrophysiological performance measures cannot readily be mapped onto each other is in line with previous research, which demonstrated that an increase in set size in the SMST did not only affect a specific ERP component, but several of them simultaneously (Schubert et al., 2015).

Because there was no linear increase in P3 latencies across set sizes, we did not compute a slope parameter that would have reflected individual differences in this increase. Similarly, there was no significant relationship between the experimental latent variable P3 and intelligence in our FLM with factor loadings fixed to 1, 3, and 3. This indicates that more and less intelligent individuals do not differ in their P3 latency trajectories. Only the association between the non-experimental latent variable (the latent intercept) and the latent variable *g* reached significance underlining that more intelligent individuals display shorter P3 latencies in general.

In our analyses, the ERP components were time-locked to the probe as we focused on the aspect of the stimulus evaluation that reflects the memory scanning. Since we could not find support for the assumption that the P3 latency reflects the same cognitive process as RTs, it would be interesting to investigate latency effects when time-locked to the memory set rather than the probe. This would allow to study processes that are related to the encoding rather than to the scanning of the memory set. Houlihan (1998) found that, similarly to increases of the P3 latency to the probe, P3 latencies to the memory increased with an increasing set size. Interestingly, more intelligent individuals displayed longer latencies than less intelligent individuals, which indicates that they devote more time to the encoding and then profit from a quicker memory scanning and recall. It would be worthwhile to apply the FLM to the context of encoding in future research to investigate whether this process might be more relevant to general intelligence than the process captured in the P3 latency time-locked to the probe.

### Limitations

Several limitations must be taken into consideration. Most importantly, Kretzschmar and Gignac (2019) highlighted the importance of sample size in order to estimate a stable correlation in a latent variable framework. For a latent correlation of *r* = |.30| and latent variables with a reliability ranging from ω = .80 to ω = .90 (in our present study ω = .87 for the experimental latent variable RT and ω = .85 for the latent variable *g*), a sample size between *N* = 250 and *N* = 340 would have been needed to estimate a latent correlation with a confidence interval of 80% (Kretzschmar & Gignac, 2019). The relatively small sample size in the present data (*N* = 102 for RTs) results in the difficulty of precisely estimating the size of the relationship between the latent constructs, and thus, it is likely that the FLM approach did not yet exploit its maximum potential to this point. In the future, it should be tested again with a larger sample size.

Another limitation concerns the question of linearity. In the present data, only three set size conditions were realized, leading to a restriction of range of the predictor variable set size. This could be an explanation for the inability to demonstrate a linear increase with set size, especially for the P3 latency data. In this context, it is interesting to consider possible floor and ceiling effects of linearity. Of course, it is always possible to fit a line through two points (i.e. two set size conditions) as for example in Ergen et al. (2012). However, with only two set size conditions realized, it is merely possible to describe the trend of the trajectory of P3 latencies regressed on set size. More data points are needed in order to be able to predict the P3 latency at different set size conditions. The more data points, the better the prediction will be. The ambiguity between the findings of this study and the research of Houlihan (1998) (an overall increase of P3 latencies with set size was found) and Pelosi et al. (1995) (an increase of P3 latencies with set size was found but only the increase from set size 1 to set size 3 reached the level of significance) could be due to the limited number of set size conditions in all of these studies. A low sampling rate of set sizes (all of these studies recorded ERP latencies only in set sizes 1, 3, and 5) makes it difficult to properly describe the function relating ERP latencies to memory set sizes. It would be interesting to explore not only the effect of set sizes left out in the present study (i.e., set sizes 2 and 4), but also the effects of larger set sizes on P3 latencies. It should be kept in mind, however, that set sizes too large to be simultaneously held in short-term memory may result in the recruitment of additional neurocognitive processes, resulting in disproportional increases in RTs and P3 latencies. Alternatively, extremely large set sizes may result in no further increase in RTs and P3 latencies, as participants may no longer be able to conduct a full search of their (overtaxed) short-term memory (Sternberg, 1969).

## Conclusions

We found support for earlier findings that RTs in the SMST increase linearly with set size, that RTs are negatively correlated with *g*, and that the slope of the linear function of RTs regressed on set size is correlated with *g*. This relationship could be increased substantially using a FLM approach. No support for the idea that P3 latencies increase linearly with set size was found, however, an increase from set size 1 to set size 3 was found. The different trajectories of RTs and P3 latencies suggest that they do not both measure the serial memory scanning in the sense of Sternberg (1969). The results suggest that the process captured by P3 latencies that takes place during the SMST is less affected by the experimental manipulation. Still, the speed of the specific process of comparing the probe stimulus with every member of the memory set (reflected in the slope of RTs regressed on set size) could be identified as the feature of a specific process that differs in efficiency between more and less intelligent individuals. Most importantly, we demonstrated that the FLM approach is undoubtedly suitable for predicting *g* by decomposing individual differences in specific experimental processes such as the scanning of information in short-term memory. The FLM could overcome deficiencies of the slope on a manifest level (low reliability and negative correlation with the intercept) and is therefore more appropriate to investigate associations between experimental slope measures and intelligence than manifest approaches.

## Data Accessibility Statements

All data are available online on this paper’s project page on the Open Science Framework at https://osf.io/q4xc8/ (https://10.17605/OSF.IO/Q4XC8).

## Additional file

Analysis scripts are available online on this paper’s project page on the Open Science Framework at https://osf.io/q4xc8/ (https://10.17605/OSF.IO/Q4XC8).
